# High-precision measurements of the hyperfine structure of cobalt ions in the deep ultraviolet range

**DOI:** 10.1038/s41598-023-31378-1

**Published:** 2023-03-23

**Authors:** Á. Koszorús, M. Block, P. Campbell, B. Cheal, R. P. de Groote, W. Gins, I. D. Moore, A. Ortiz-Cortes, A. Raggio, J. Warbinek

**Affiliations:** 1 Department of Physics, University of Liverpool, Liverpool, L69 7ZE United Kingdom; 2 GSI Helmholtzzentrum für Schwerionenforschung GmbH, 64291 Darmstadt, Germany; 3 Helmholtz Institute Mainz, 55099 Mainz, Germany; 4 Department of Chemistry - TRIGA site, University of Mainz, 55099 Mainz, Germany; 5 Department of Physics and Astronomy, University of Manchester, Manchester, M13 9PL United Kingdom; 6 Department of Physics, University of Jyväskylä, PB 35(YFL), 40351 Jyväskylä, Finland; 7 Grand Accélérateur National d’Ions Lourds (GANIL), CEA/DSM-CNRS/IN2P3, Caen, France; 8 Present Address: Experimental Physics Department, CERN, CH1211 Geneva 23, Switzerland; 9 Present Address: Instituut voor Kern- en Stralingsfysica, KU Leuven, 3001 Leuven, Belgium

**Keywords:** Atomic and molecular interactions with photons, Optical spectroscopy

## Abstract

High-precision hyperfine structure measurements were performed on stable, singly-charged $^{59}$Co ions at the IGISOL facility in Jyväskylä, Finland using the collinear laser spectroscopy technique. A newly installed light collection setup enabled the study of transitions in the 230 nm wavelength range from low-lying states below 6000 cm$^{-1}$. We report a 100-fold improvement on the precision of the hyperfine *A* parameters, and furthermore present newly measured hyperfine *B* paramaters.

## Introduction

The study of the hyperfine structure (hfs) of atomic spectra is of great importance for several fields ranging from astrophysics^1–3^ and nuclear physics^4^ to metrology^5,6^. The iron group elements, in particular, are abundant in solar and stellar spectra, and play an important role in understanding stellar evolution, galactic chemical evolution and nucleosynthesis, as well as variations of fundamental constants^7–10^. It has been shown that accurate knowledge of the hyperfine structure constants and isotope shifts is essential for analysing astrophysical spectra^1^, and neglecting these can lead to inaccurate results. Thus, great progress has been made recently to extend the list of known atomic parameters^7,11,12^. While the magnetic dipole interaction constant *A* is known for over 86 observed energy levels in the cobalt ion (Co II) with reasonable precision, the electric quadrupole interaction constant *B* could only be estimated for one level so far due to the low spectral resolution of the obtained spectra^11^. In contrast, the hyperfine *A* and *B* parameters of neutral Co (Co I) are known for over 330 and 150 lines respectively, including precise measurements of the hyperfine splitting of low-lying states using atomic-beam magnetic resonance, where the accuracy of the *A* constants was obtained with better than 0.0005% precision^13,14^.

**Figure 1:**
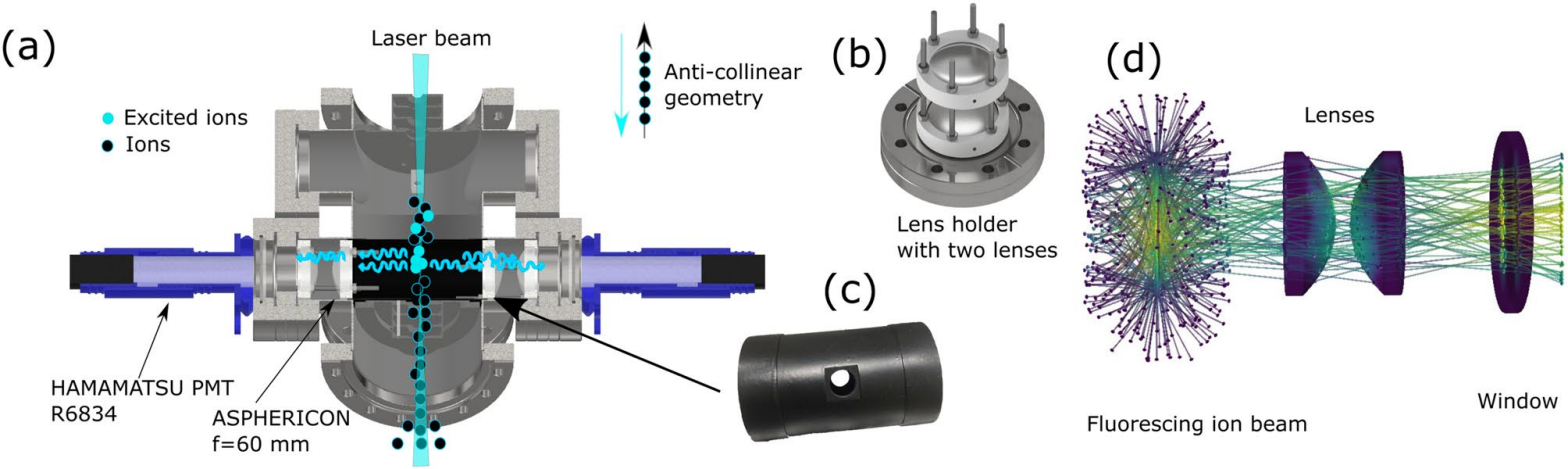
The light collection chamber installed for the measurement of Co II. (**a**) Custom-made chamber with 4 windows for light collection. The length is 34 cm and the distance between the two rows for the optical detection system is 12 cm. (**b**) Lens assembly for imaging the ion beam onto a photomultiplier tube. For different wavelengths, the position of the lenses can be optimized for maximum efficiency. (**c**) Black cylinder for minimizing the background rate due to scattered laser light. (**d**) Simulation of the light collection systems using the Raosi python package. This algorithm is used to obtain the optimal distance of the lenses and PMT for a given wavelength.

Collinear laser spectroscopy techniques enable precise measurements of the hyperfine structure of a wide range of elements, and are widely used in radioactive ion beam facilities for the study of short-lived nuclei^4^. These experiments aim to extract the nuclear electromagnetic moments and changes in the mean-squared charge radii of nuclei far from $\beta$-stability and contribute to our understanding of the nuclear forces^4^. Cobalt is a chemical element with $Z=27$ protons. The unstable isotopes of Co have so far not been explored using laser spectroscopy techniques, despite great nuclear structure interest. These isotopes would, for example, allow for the investigation of nuclear phenomena such as nuclear deformation and single-particle nature in the vicinity of the nuclear ‘magic’ number $Z=28$, and the isospin symmetry using the self-conjugate $^{54}$Co isotope^15^. Furthermore, the electromagnetic moments and the nuclear charge radii of a proton-emitting nuclear isomer like $^{53m}$Co studied in Refs.^16,17^ have never been measured before, and thus presents a compelling case for laser spectroscopy experiments. Before attempting to perform laser spectroscopy measurements on weakly produced short-lived isotopes, it is crucial to explore the sensitivity of the hfs to the nuclear properties using the stable isotope $^{59}$Co, i.e., to measure the magnetic dipole interaction constant *A* and the electric quadrupole interaction constant *B*. In addition, the atomic field- and mass-shift constants of the energy levels are of utmost importance to investigate the experimental sensitivity to the changes in the nuclear charge radii. However, these factors cannot be investigated by performing measurements on beams of naturally occurring Co, as it only has one stable isotope. Thus, the atomic field- and mass-shift constants have to be extracted from theoretical calculations^18^. The results presented in this work motivate the development of theoretical methods and can be used to benchmark these calculations in the future.

We report precise laboratory measurements of the hyperfine parameters in the Co ion in the 230 nm range, starting from the energy levels in the $3d^7(^4$F)4*s* configuration. Besides the newly measured hyperfine *B* constants, the precision of the known hyperfine *A* constants was improved by a factor of 100.

## Experimental methodology

Collinear laser spectroscopy^4,19^ was performed at the Ion Guide Separator On-Line (IGISOL) IV facility, located at the University of Jyväskylä JYFL Accelerator Laboratory, Finland^20^. Singly-charged Co ions were produced in a discharge ion source in an environment of a helium buffer gas at a pressure of $\sim$25 mbar. Once extracted from the ion source in a gas jet, through a sextupole ion guide, and passing through a differential pumping section, the ions were formed into a 30 keV beam which was subsequently mass separated using a dipole magnet with a resolving power of $\frac{m}{\Delta m}=$300. The beam could then be cooled and bunched using a linear gas-filled radio-frequency quadrupole Paul trap (cooler-buncher)^21^, from which bunches with a temporal width of 10 $\upmu$s were released every 100 ms and directed to the laser spectroscopy station. This is advantageous with low beam currents (order of $10^6$ particles per second or less) in the case of unfavorable signal-to-noise ratios. However, most of the measurements reported here were taken with a continuous ion beam since sufficient ion currents were available. Nevertheless, the ions still passed through the Paul trap for cooling to room temperature. In combination with the acceleration to 30 keV, this results in narrow spectral linewidths, with the residual Doppler broadening being comparable to the natural linewidth. The ions were then overlapped with a laser beam in an anti-collinear geometry as shown in Fig. 1a. Between 0.1 mW and 1.5 mW of ultraviolet light was produced by frequency-quadrupling a continuous-wave narrow-linewidth Sirah Matisse TS Ti:Sa laser. This was achieved by placing two Sirah Wavetrain frequency doubling cavities in series. Due to the humidity in the air, the production of laser light in the fundamental 940 nm range can be challenging, as this coincides with the absorption spectrum of H$_{2}$O molecules, which are abundant in the air. To ensure a stable operation of the laser system, the laser cavity and the first frequency doubling cavity were purged with nitrogen gas.

For the study of transitions in Co II, a new light collection-region (LCR) was designed and installed, shown in Fig. 1. The full length of the chamber is 34 cm and it is equipped with four ports for light collection. In this experiment, only the first two ports were equipped with PhotoMultiplier Tubes (PMTs), as shown in Fig. 1a, and the second row was closed with blank flanges. A turbo pump is installed in the bottom of the chamber to ensure ${10^{-8}}$ mbar vacuum in the light-collection region which is necessary to reduce the collisional excitation rate with the residual gas. In order to measure the hyperfine structure of the Co isotopes, which spans across a frequency range of up to 30 GHz, the Doppler tuning technique was used, where instead of changing the laser frequency, the velocity of the ion beam is adjusted to bring it on resonance with different ionic transitions. To achieve this, a voltage is applied to the whole chamber, which thus needs to be electrically isolated from the rest of the beamline. The ions are shielded from the high potential of the chamber while they fly through the first apertures installed at the beginning of the chamber. Once the ions reach the final aperture they are accelerated as required and interact with the laser beam in front of the optical detection system. To ensure good alignment of the laser and ion beam, a 1 mm diameter aperture can be inserted during the ion beam tuning step.

The light-collection system consists of 2 aspherical lenses manufactured by Asphericon (AFL50-60-S-U) with 50 mm diameter and 60 mm focal length, presented in Fig. 1b. These lenses have good transmission properties in the UV wavelength range. The Hamamatsu R6834 PMTs were mounted from the outside and are electrically isolated from the chamber. The PMTs were selected to have a low dark count rate, of the order of 1–5 counts per second. The PMTs are biased using a C14019 socket, which also contains a signal amplifier. The background rate due to scattered laser light is minimized using a set of apertures on both the incoming and outgoing beam, and by painting the inside of the chamber with Aquadag graphite (G303E). In addition, a cylinder was installed in front of the light-detection assembly, perpendicular to the axis of the laser and ion beam, with two holes allowing both ions and laser beams to pass through, shown in Fig. 1c. This further reduces the background rate, resulting in a total background rate of 1000 counts per second per mW of laser power at 232 nm, in the two PMTs combined. With the typical bunch length of 10 $\mu$s, in bunched-beam laser spectroscopy with 10 Hz duty cycle, this would result in a 0.1 counts per second background rate. The optimal position of the lenses and the PMTs for a given wavelength were calculated using the Raosi python ray-tracing package, developed by our group for this purpose^22^. It allows for the selection of the material of the windows and the lenses, taking their optical properties into account accurately. Laser spectroscopy of neutral atoms is also possible using this setup, as reported in Ref.^23^.

The signal from the two PMTs is directly connected to a Cronologic TimeTagger 4–2G Time-to-Digital-Converter (TDC). The TDC has four channels, each with a 500 ps single-shot resolution. To generate the voltage for Doppler tuning the ions, the 16-bit analog output of a Measurement Computing USB-3102 data acquisition board is used. The low voltage from this device, − 10 V to 10 V, is amplified by a $\times$ 1000 TREK 609E-6 high-voltage amplifier, with a slew rate of 150 V/$\upmu$s. This high voltage is applied to the light-collection chamber presented in Fig. 1a). The settling time for 5 kV voltage changes was determined to be less than 200 $\upmu$s. Before every hfs spectrum measurement a voltage calibration is performed using a Keysight 34465 digital multimeter, which reads out the applied voltage using a 1:1000 voltage divider. The platform potential of the cooler voltage is also measured using another Keysight 34465 multimeter and a KV-30A 1:10000 voltage divider from Ohm-Labs. For a typical measurement, the voltage step is between 0.5 and 2 V, and the voltage change is synchronized to the bunch release of the cooler. The laser frequency was locked to the Doppler-shifted transition frequency of the studied transition using a HighFinesse WSU/10 wavelength meter. The values for these transitions in the rest-frame of the ions were taken from the NIST database^24^. For every measurement 2 CSV data files are recorded. One contains the laser frequency, the readout of the cooler-buncher potential, and then a calibration of the voltages applied to the LCR. The second file contains one row of data for every event detected by the TDC, which logs the scanning voltage setpoint and the TDC timestamp.

The measured transitions in Co II are in the 228–232 nm range and have transition strengths of the order of $A=10^8$ s$^{-1}$. Five transitions have been studied in Co II, from the metastable states with a $3d^7(^4F)4s$ configuration to states with a $3d^7(^4F)4p$ configuration. The lower-lying energy levels are well populated when the ions are produced in a spark-discharge source, and also survive the collisions with the He buffer gas in the cooler-buncher device. The advantage of these transitions with respect to those from the ground-state is that the configuration provides higher sensitivity to the nuclear properties as it involves the excitation of an *s* electron. The transitions from the ground-state could not be investigated at this time, as they require a different wavelength. The difference between the relative population of these two configurations due to the discharge source condition is therefore unknown. Optical pumping in the cooler-buncher device may be utilized in the future to increase the population in the metastable states by pumping the electrons from the ground state^20^.

**Figure 2:**
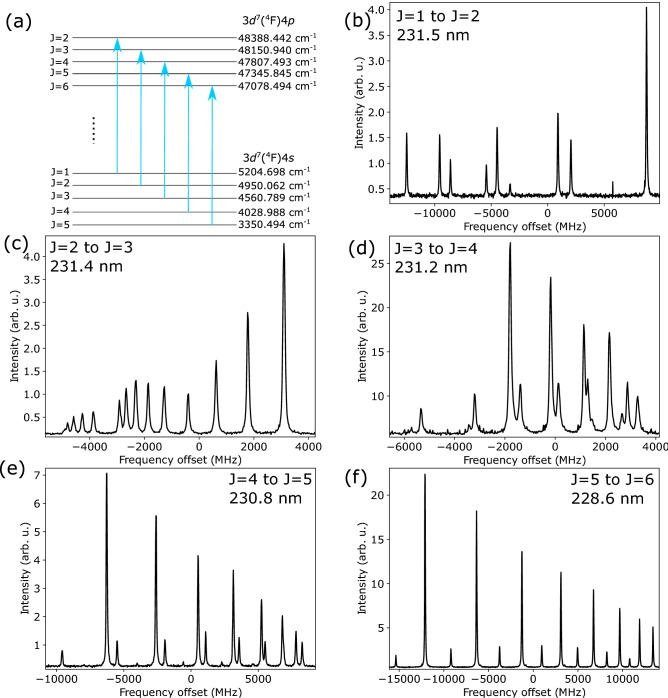
(**a**) Measured transitions in singly ionized Co. The extracted hyperfine parameters are presented in Table 1 separately for each level. (**b**–**f**) The measured hyperfine structure spectrum of transitions indicated in figure (**a**). The frequency axis is provided with an arbitrary offset. The intensity corresponds to $10^{-3}$ times the number of detected photons during the measurement.

**Table 1:** Information on the energy levels and the measured hyperfine constants of $^{59}$Co II ($I=$ 7/2). The *A* parameters are compared to literature^12^.

*E* (cm$^{-1}$)	*J*	$A_{Lit}$ (MHz)	*A* (MHz)	*B* (MHz)	$A/\mu$ (MHz/$\mu _{N}$)	*B*/*Q* (MHz/b)	Fig. 2
3350.494	5	1031 (30)	1056.76 (21) [23]	132 (10)	228.4 (4)	314 (33)	f
4028.988	4	840 (50)	925.94 (13) [20]	97 (5)	200.1 (4)	231 (20)	e
4560.789	3	760 (70)	788.29 (20) [17]	47 (7)	170.37 (33)	112 (18)	d
4950.062	2	510 (60)	551.64 (18) [12]	32 (2)	119.922 (24)	76 (7)	c
5204.698	1	$-$ 250 (40)	$-$ 261.42 (24)	31.2 (10)	-56.50 (12)	74 (6)	b
47078.494	6	330 (60)	333.52 (14) [7]	264 (5)	72.08 (14)	630 (50)	f
47345.845	5	390 ( (60)	380.45 (14) [8]	245 (8)	82.22 (16)	580 (50)	e
47807.493	4	500 (60)	460.59 (16) [10]	201 (4)	99.54 (20)	479 (35)	d
48150.940	3	600 (40)	659.53 (13) [13]	178.0 (22)	142.54 (28)	424 (31)	c
48388.422	2	1196 (30)	1198.46 (20) [25]	174.4 (28)	259.9 (5)	415 (30)	b

Statistical uncertainties are reported in brackets and systematic uncertainties are reported in square brackets. In the sixth and seventh column the hyperfine field constants are reported. The last column indicates the figure of the measured spectrum in Fig. 2.

## Data analysis

Since the measurements were performed on a fast ion beam, the measured frequencies $\nu$ in the spectra were first converted into the rest-frame of the ions $\nu _{R}$, using the following equation:

1 $$\begin{aligned} \nu _{R} = \nu \sqrt{\frac{1+\beta }{1-\beta }}, \quad \beta = \sqrt{1-\left( \frac{mc^2}{mc^2+qV}\right) ^2}, \end{aligned}$$

where *m* and *q* are the mass and the charge of the ions, V is the acceleration potential of the beam and *c* stands for the speed of light. For an atom with nuclear spin $I>0$, individual fine structure atomic energy levels with total angular momentum $J>0$ are split due to the hyperfine interaction. In general, the energies of the hyperfine levels with angular momentum *F* are shifted from the fine structure energy according to:

2 $$\begin{aligned} \Delta E = \frac{1}{2}AK+B\frac{(3/4)K(K+1)-J(J+1)I(I+1)}{2I(2I-1)J(2J-1)}, \end{aligned}$$

where $K=F(F+1)-J(J+1)-I(I+1)$. Note that the second term is only present for both $I,J>1/2$. The relative intensities of the hyperfine transitions from the lower states with $F_{L}$, $J_{L}$ to the upper states, $F_{U}$, $J_{U}$, can be obtained as follows:

3 $$\begin{aligned} R(F_{U},F_{L}) = (2F_{U}+1)(2F_{L}+1)\begin{Bmatrix} I&\quad F_{U}&\quad J_{U}\\ 1&\quad J_{L}&\quad F_{L} \end{Bmatrix}, \end{aligned}$$

where the last term is the 6-j symbol. In collinear laser spectroscopy experiments the intensity of the hyperfine peaks is typically in excellent agreement with Eq. (3). When the low spectral resolution prevents the separation of individual peaks, Eq. (3) is used to fix these parameters in the fitting procedure. In this work, the obtained results of the analysis with free and fixed intensities were identical. If the nuclear electromagnetic moments are known, the hyperfine fields can be extracted for the measured hfs and vice versa using:

4 $$\begin{aligned} A=\frac{\mu B(0)}{IJ}, \qquad B=eQV_{zz} , \end{aligned}$$

where *A* and *B* are the earlier introduced hyperfine parameters, *e* the electric charge, *B*(0) is the magnetic field at the site of the nucleus, $V_{zz}$ the electric field gradient generated by the electrons at the nucleus, and finally $\mu$ and *Q* are the nuclear magnetic dipole and electric quadrupole moments, respectively.

The presented measurements were performed in the same experiment on the same day. The spectra of the transitions presented in Fig. 2b–f were recorded 4, 2, 4, 3 and 2 times, respectively. The measured hyperfine structure spectra were fitted using the SATLAS package^25^ and the hyperfine *A* and *B* constants were extracted from this fit. The spectral lines were modeled using a Voigt profile, with a typical linewidth of around 85 MHz full width at half maximum. In addition to the $\chi ^2$ fitting, a random walk was performed to map the correlations between the hyperfine parameters, as explained in Ref.^25^. The results of the $\chi ^2$ fit and random walk analysis were consistent, with comparable values for the uncertainties. From each measurement the hyperfine structure parameters *A* and *B* were extracted for both upper and the lower electronic state, as well as the centroid of the structure. Given that these 5 parameters are extracted from the analysis while fitting more than 8 peaks, the system of equations is always overdetermined. The final results were obtained by calculating the weighted mean of the results of the individual measurements.

## Results and discussion

The transitions measured in singly ionized Co in this work are depicted in Fig. 2. The nuclear spin of $^{59}$Co is $I=$7/2. The energy levels within the $3d^7(^4$F)4*s* configurations were well populated in the discharge ion source and the hfs could be obtained with high statistical precision. From the estimated efficiency of different lines, the lowest energy level (with $J=5$) was the most populated state. The span of the full hfs varies between 8 GHz and 30 GHz, with the $J=5$ to $J=6$ transition covering the largest range.

Information on the levels and their extracted hyperfine *A* and *B* constants are presented in Table 1. In addition to a statistical uncertainty, the reported final results also include an uncertainty due to the energy of the ion beam. This has been explicitly added in square brackets for the cases in which this uncertainty is not negligible compared to the statistical uncertainty. The absolute voltage which is used to accelerate the ions when they exit the cooler-buncher is only known to an accuracy of 10 V, estimated based on previous calibration measurements using the same setup, as described in Ref.^23^. This may result in a slight stretching of the hfs spectrum during the conversion from the laboratory frame to the rest frame of the atoms. The total energy of the measured transitions is not reported here, since the experiment focuses on differential changes. The hyperfine *A* constants are compared to literature^12^, and are found to be in excellent agreement with earlier measurements. In this work we have improved the precision by a factor of 100. Hyperfine *B* constants were also obtained for the first time. In previous works using Fourier transform spectrometry, these *B*-constants were consistent with 0^11,12^. The uncertainty of the extracted *B* is significantly higher than for *A*. This can be attributed to strong correlations between the *B* parameters of the lower and upper hyperfine energy levels. Given the high precision of the hyperfine *A* parameters, second-order hyperfine effects may need to be assessed to ensure the accuracy of these values. However, these effects are expected to be smaller than the final uncertainty of the results.

The hyperfine field coefficients were extracted from the measured *A*/$\mu$ and *B*/*Q* using Eq. 4. The nuclear magnetic dipole moment, $\mu =+4.615(25)$ $\upmu _{N}$ was taken from Ref.^26^. It was obtained from nuclear magnetic resonance studies of intermetallic compounds. For the nuclear electric quadrupole moment, the value $Q=0.4$2(3) b was used from Ref.^27^. These extracted hyperfine field values are presented in Table 1 for every level measured in this work.

## Conclusions

The hyperfine structure of five transitions in Co II was measured from the low-lying levels with $3d^7(^4$F)4*s* configuration in the 230 nm range. The hyperfine *A* parameters could be extracted with high precision from the obtained spectra, improving the precision by two orders of magnitude compared to literature. The hyperfine *B* constants, measured for the first time in this work, are also presented, increasing the number of reported *B* parameters in Co II to 12. Their uncertainty is considerably larger than for the *A* constants, but is nevertheless sufficient for future precision measurements of electric quadrupole moments of unstable cobalt isotopes. While theoretical calculations of the atomic parameters for the Co atom are available^28^, the same is not true for the singly-charged ion, to our knowledge. A comparison to the predictions of atomic theory was therefore not possible.

A low background rate and excellent signal-to-background ratio in the measured spectra was enabled by the newly installed light-collection region developed specifically for this work. The presented results demonstrate that the measurement of weakly produced radioactive Co isotopes is now possible at the IGISOL laboratory using the experimental setup and transitions presented in this work.

## Data Availability

All data generated or analysed during this study are included in this published article.
